# Cataloguing and Selection of mRNAs Localized to Dendrites in Neurons and Regulated by RNA-Binding Proteins in RNA Granules

**DOI:** 10.3390/biom10020167

**Published:** 2020-01-22

**Authors:** Rie Ohashi, Nobuyuki Shiina

**Affiliations:** 1 Life Science Research Center, University of Toyama, Toyama 930-0194, Japan; 2 Laboratory of Neuronal Cell Biology, National Institute for Basic Biology, Okazaki, Aichi 444-8585, Japan; 3 Department of Basic Biology, SOKENDAI, Okazaki, Aichi 444-8585, Japan; 4 Exploratory Research Center on Life and Living Systems, Okazaki, Aichi 444-8585, Japan

**Keywords:** local translation, dendritic mRNA, RNA-binding proteins, RNA granules

## Abstract

Spatiotemporal translational regulation plays a key role in determining cell fate and function. Specifically, in neurons, local translation in dendrites is essential for synaptic plasticity and long-term memory formation. To achieve local translation, RNA-binding proteins in RNA granules regulate target mRNA stability, localization, and translation. To date, mRNAs localized to dendrites have been identified by comprehensive analyses. In addition, mRNAs associated with and regulated by RNA-binding proteins have been identified using various methods in many studies. However, the results obtained from these numerous studies have not been compiled together. In this review, we have catalogued mRNAs that are localized to dendrites and are associated with and regulated by the RNA-binding proteins fragile X mental retardation protein (FMRP), RNA granule protein 105 (RNG105, also known as Caprin1), Ras-GAP SH3 domain binding protein (G3BP), cytoplasmic polyadenylation element binding protein 1 (CPEB1), and staufen double-stranded RNA binding proteins 1 and 2 (Stau1 and Stau2) in RNA granules. This review provides comprehensive information on dendritic mRNAs, the neuronal functions of mRNA-encoded proteins, the association of dendritic mRNAs with RNA-binding proteins in RNA granules, and the effects of RNA-binding proteins on mRNA regulation. These findings provide insights into the mechanistic basis of protein-synthesis-dependent synaptic plasticity and memory formation and contribute to future efforts to understand the physiological implications of local regulation of dendritic mRNAs in neurons.

## 1. Introduction

Spatiotemporal translational regulation is key to increasing the concentration of specific proteins to exert their functions at specific timings and locations in cells. In neurons, mRNAs are translated not only in the cell soma but also in axons and dendrites [1,2]. Local translation in axons is mainly required for axon outgrowth and maintenance, and local translation in dendrites is necessary for synaptic plasticity and long-term memory formation [1,2,3,4].

In 1996, Kang and Schuman revealed that long-term potentiation (LTP) occurs in the stratum radiatum (SR), which is a dendrite-enriched region in hippocampal CA1, even after the SR was isolated from the cell-soma-enriched stratum pyramidale (SP). LTP in the isolated SR was lost after inhibition of protein synthesis [5]. Their findings suggest the existence of “local translation” in neurites, and that proteins translated locally in neurites are sufficient for LTP induction. Since these findings, our understanding of local translation in dendrites has deepened substantially: specific mRNAs are recruited to “RNA granules”, which are membrane-less RNA–protein complexes containing mRNAs, RNA-binding proteins, ribosomes, and translational regulators, and are transported to dendrites using microtubules as rails [6,7,8]. RNA-binding proteins in RNA granules are key regulators of mRNA localization and protein synthesis. Dysfunction of these proteins causes abnormalities in dendritic mRNA localization and translation, resulting in impairment of higher-order brain functions, such as neurodevelopmental disorders, intellectual and mental disorders, and loss of long-term memory formation [9,10].

This review catalogues and selects mRNAs that are localized to dendrites and regulated by RNA-binding proteins in RNA granules. Here, we focus on dendritic mRNAs that have been identified in common in several studies that identified dendritic mRNAs based on various criteria using comprehensive RNA sequencing (RNA-seq) of the SR from rodent hippocampal CA1. These mRNAs are further classified into groups according to the functions of the mRNA-encoded proteins. In addition, we focus on the major RNA-binding proteins in RNA granules and list their target mRNAs and effects on mRNAs. Furthermore, we compared our selected set of dendritic mRNAs with the RNA-binding protein target mRNAs and discuss the contribution of RNA-binding proteins to mRNA regulation, such as mRNA expression, stability, localization, and translation, for the regulation of local translation in dendrites.

## 2. mRNAs That Are Localized in the Dendrite-Enriched Layer of the Hippocampus

**Figure 1:**
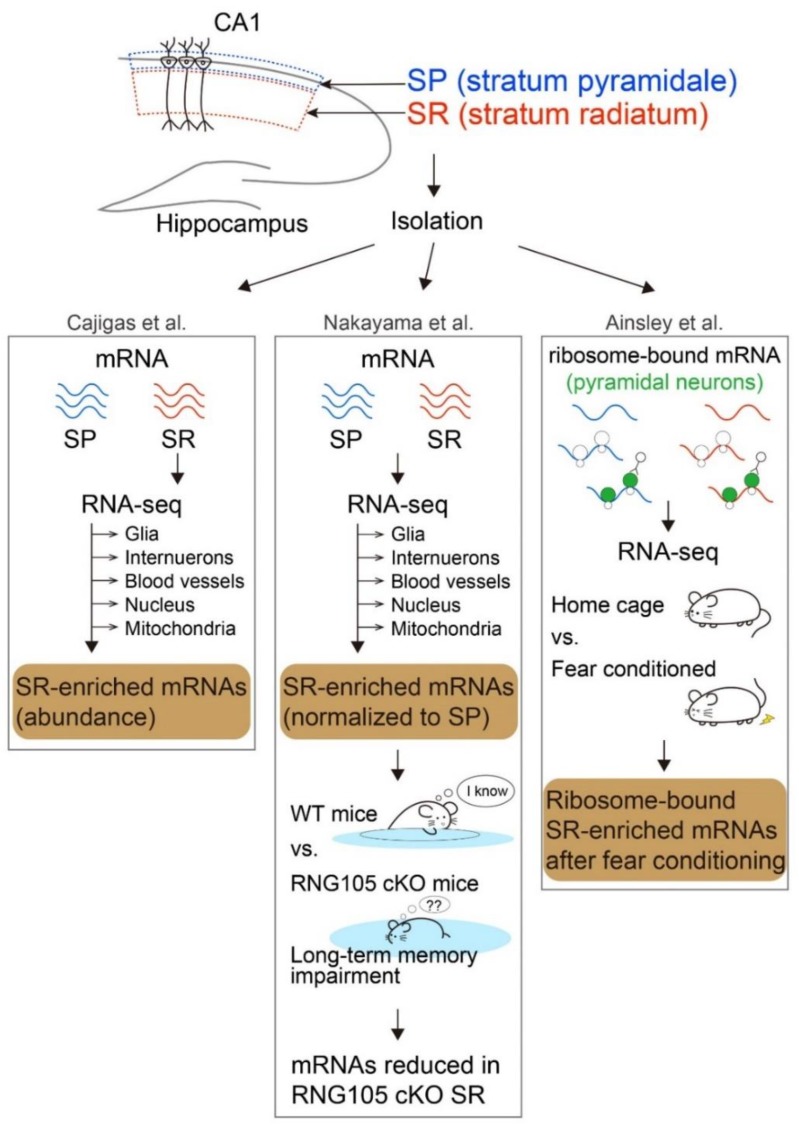
Identification of mRNAs localized to the dendrite-enriched stratum radiatum (SR) layer in hippocampal CA1. Three different groups comprehensively identified mRNAs localized in the hippocampal SR layer using next-generation RNA sequencing. In the hippocampus, somas align in the stratum pyramidale (SP) and dendrites elongate into the SR. Cajigas et al., Nakayama et al., and Ainsley et al. identified mRNAs localized to the hippocampal SP and SR layers after isolating the layers from rodents. Cajigas et al. identified mRNAs abundant in the SR of the rat hippocampal CA1 region. Nakayama et al. identified mRNAs that are more enriched in the SR layer compared with the SP layer in the mouse hippocampus. They also identified mRNAs that were localized to the SR but were reduced in the SR of RNA granule protein 105 (RNG105, also known as Caprin1) conditional knockout (cKO) mice that showed long-term memory impairment. Ainsley et al. identified ribosome-bound mRNAs in the hippocampal SR of fear-conditioned mice. In this review, we compared the SR-enriched mRNA lists from these studies (colored in brown) and focused on dendritic mRNAs identified in common in all of these studies (Supplementary Table S1).

In the 1990s, several mRNAs were identified as dendritically localized mRNAs, among which *Camk2a*, *Map2*, *Arc*, and *Insp3r1* mRNAs were intensively studied [11,12]. Later, with the help of advances in deep sequencing technology, thousands of mRNAs that are localized to dendrites have been identified from the dendrite-enriched layer (SR) in the rodent hippocampus [13,14,15,16,17]. We briefly review studies that comprehensively identified the candidates of dendritic mRNAs from the SR of hippocampal CA1 using RNA-seq (Figure 1) and focus on selective mRNAs identified in common among these studies.

### 2.1. mRNAs That Are Identified in SR Isolated from Rodent Hippocampal CA1

Cajigas et al. identified mRNAs that are abundant in the synaptic neuropil in the rat hippocampus (Figure 1) [15]. They microdissected the SR and stratum lacunosum moleculare (SLM) as the neuropil layer from the hippocampal CA1 region of adult rat brains and subjected them to RNA-seq. From the raw dataset of RNA-seq, they subtracted mRNAs enriched in various types of cells other than pyramidal neurons, such as glial cells, interneurons, blood vessels, nuclei, and mitochondria, and identified 2550 transcripts as dendritic and/or axonal mRNAs. Subsequent mRNA labeling with high-resolution in situ hybridization revealed that all 71 mRNAs for which the authors developed probes were detected in dendrites, suggesting that most of the mRNAs identified from the SR and SLM layers using this method are dendritic mRNAs.

Nakayama et al. identified mRNAs more enriched in the SR layer compared with the SP layer in the hippocampus of adult mice (Figure 1) [16]. They microdissected and isolated the SP and SR layers from mouse hippocampal CA1 and subjected them to RNA-seq. From the RNA-seq raw data, as in the study of Cajigas et al., mRNAs enriched in various cell types other than pyramidal neurons were subtracted. Relative read counts of transcripts in the SR compared with the SP were calculated, which identified 2106 SP-enriched mRNAs and 1122 SR-enriched mRNAs as somatically and dendritically enriched mRNAs, respectively. The authors also revealed that the enrichment of SR-enriched mRNAs in the SR layer was reduced by conditional knockout (cKO) of RNA granule protein 105 (RNG105, also known as Caprin1), deficiency of which severely impaired long-term memory formation.

Ainsley et al. identified ribosome-bound mRNAs in the hippocampus of mice that had received a novel experience causing activity-induced memory formation (Figure 1) [17]. They collected tissues from the SP and SR layers of the hippocampal CA1 of adult mice expressing EGFP-tagged ribosomal protein L10a (Rpl10a) specifically in pyramidal neurons but not in glia or interneurons. SP- and SR-enriched mRNAs bound to EGFP-tagged ribosomes were obtained by immunoprecipitation and then sequenced using RNA-seq. The RNA-seq data from resting mice and mice exposed to a novel experience consisting of a contextual-fear-conditioning trial were analyzed by machine learning classification, which listed 1860 ribosome-bound dendritically localized mRNAs after fear conditioning. Because mRNA binding to ribosomes increases during translation, these ribosome-bound mRNAs were thought to be translated locally in dendrites in an activity-dependent manner.

**Figure 2:**
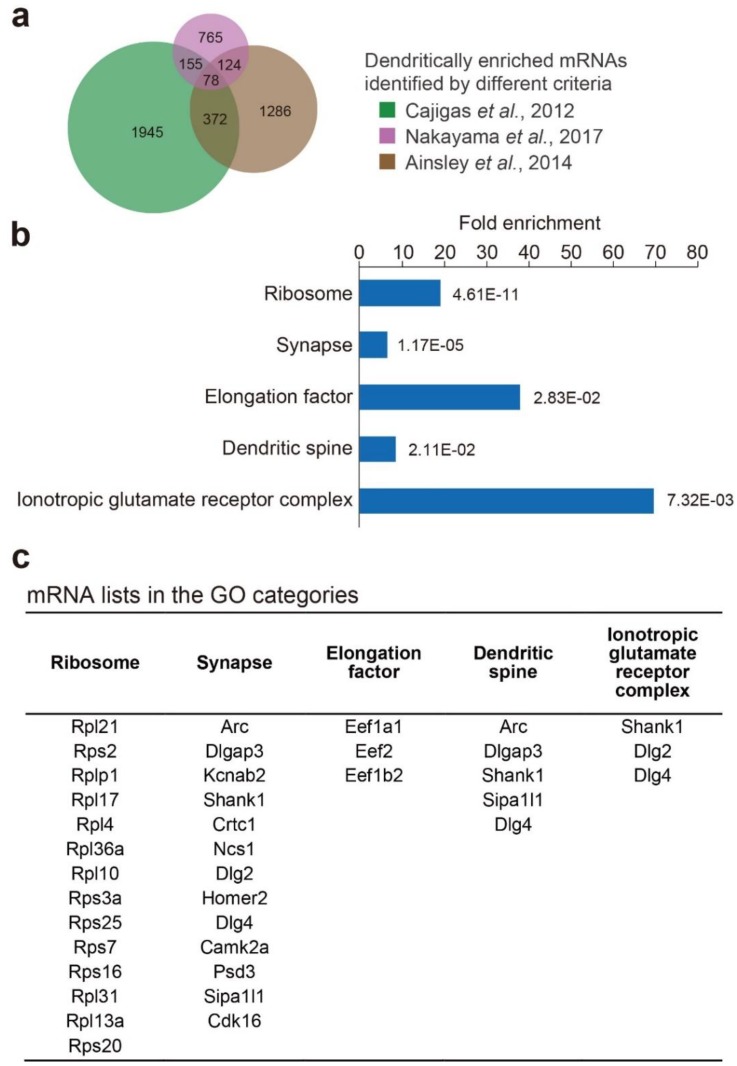
Gene ontology (GO) categories in which the identified dendritic mRNAs were enriched. The three studies (Cajigas et al., 2012; Ainsley et al., 2014; Nakayama et al., 2017) were compared, and 78 mRNAs were found to be commonly identified in the studies (**a**). The 78 mRNAs were classified by GO enrichment analysis using DAVID 6.8. GO categories in which the mRNAs were significantly enriched (**b**), and the list of mRNAs included in the GO categories (**c**) are shown.

These three studies used different strategies to identify dendritic mRNAs in term of abundance, relative enrichment, and ribosome binding. In particular, the strategy used by Ainsley et al. differed significantly from the other two studies in that they used transgenic mice overexpressing Rpl10a and analyzed selective mRNAs that bound to ribosomes. Due to this difference, it may be supposed that the overlap of dendritic mRNAs between Ainsley et al. and the other two studies may be smaller than that between the other two. However, this was not the case (Figure 2a), suggesting that dendritic translation may not occur in a highly mRNA-selective manner. We found that the number of overlapped dendritic mRNAs among the three studies was 78, representing only 3%–7% of the total number of dendritic mRNAs identified in each study (Figure 2a, Table 1, and Supplementary Table S1). Therefore, these 78 mRNAs are considered fairly reliable candidates for mRNAs localized to dendrites and locally translated in an activity-dependent manner. Recently, Farris et al. also identified mRNAs localized in the hippocampal subregions (CA1, CA2, CA3, and the dentate gyrus) of the adult mouse hippocampus [18]. They found that 68 of the 78 mRNAs overlapped as mRNAs enriched in the SR layer of the CA1 region among their study and the three studies above [18], supporting the reliable dendritic localization of the commonly identified mRNAs.

The general functions and neuronal/brain functions of these 78 mRNA-encoded proteins are summarized in Supplementary Table S1 [19,20,21,22,23,24,25,26,27,28,29,30,31,32,33,34,35,36,37,38,39,40,41,42,43,44,45,46,47,48,49,50,51,52,53,54,55,56,57,58,59,60,61,62,63,64,65,66,67,68,69,70,71,72,73,74,75,76,77,78,79,80,81,82,83,84,85,86,87,88,89,90,91,92,93,94,95,96,97,98,99,100,101,102,103,104,105,106,107,108,109,110,111,112,113,114,115,116,117,118,119,120,121,122,123,124,125,126,127,128,129,130,131,132,133,134,135,136,137,138,139,140,141,142,143,144,145,146,147,148,149,150,151,152,153,154,155,156,157,158,159,160,161,162,163,164,165,166,167,168,169,170,171,172,173,174,175,176,177,178,179,180,181,182,183,184,185,186,187,188,189,190,191,192,193,194]. Of note, most of the 78 mRNAs encoded proteins involved in the regulation of synaptic functions, such as α-amino-3-hydroxy-5-methyl-4-isoxazolepropionic acid (AMPA) receptor trafficking and transmission, *N*-methyl-d-aspartate (NMDA) receptor activity, ion channel activity, spine growth and maintenance, synaptic plasticity, LTP, and learning and memory. In addition, their dysfunctions were associated with a variety of neuropsychiatric disorders such as fragile X syndrome, Alzheimer’s disease, Down syndrome, schizophrenia, Parkinson’s disease, and autism spectrum disorder (ASD) (Supplementary Table S1). In order to understand the major biological and functional categories in which the proteins encoded by these mRNAs are involved, they were classified by gene ontology (GO) enrichment analysis using DAVID 6.8. As a result, these mRNAs were enriched mainly in the categories of “ribosome”, “synapse”, “elongation factor”, “dendritic spine”, and “ionotropic glutamate receptor complex” (Figure 2b,c).

### 2.2. Unexpected GO Categories of the Catalogued Dendritic mRNAs: “Ribosome” and “Elongation Factor”

The fact that many mRNAs encoding ribosomal proteins were found in the dendritic mRNAs was surprising. It is well known that ribosome subunits are assembled in the nucleus, where ribosome assembly requires many steps and with the help of various regulatory factors [195]. The resulting ribosomes are recruited to RNA granules and transported to dendrites [1,7,196]. Thus, although the possibility of ribosome biogenesis in dendrites cannot be ruled out, it seems almost impossible. Another possible involvement of local translation of ribosomal proteins is ribosome heterogeneity in neurons. It has been suggested that the structure of ribosomes is often not uniform in different tissues, in cells at different developmental stages, and even in distinct subcellular locations within the same cell. Such ribosomes recognize specific mRNAs through differences in components and chemical modifications of ribosomal subunit proteins, which is known as the “ribosome filter hypothesis” [197]. For example, the components of ribosomal subunits are different between testis and liver [198], ribosomes in mouse embryonic stem cells are heterogeneous and translate distinct subpools of mRNAs [199], and changes in the composition of ribosomal proteins regulate the translation of specific mRNAs in neocortical development [200,201]. In yeast, translation of *ASH* mRNA, localized at the tip of daughter cells, requires ribosomes composed of specific ribosomal protein paralogs [202]. Given that mRNA translation differs between soma and dendrites, local translation in dendrites of mRNAs encoding ribosomal proteins could cause subcellular ribosome heterogeneity in neurons. This mechanism may require the exchange of ribosomal subunit proteins in dendrites. Ribosomal protein exchange has been reported in *Escherichia coli* in several studies [203,204,205], and notably, damaged ribosomes are repaired by exchanging ribosomal subunit proteins [206]. Thus, the exchange of ribosomal subunit proteins could occur in neurons, which may contribute not only to ribosome heterogeneity but also to the maintenance of functional ribosomes in dendrites. Another possibility is that ribosomal subunit proteins have their own functions by themselves. The physiological relevance of the localization of ribosomal-protein-encoding mRNAs to dendrites is unknown, but it is very interesting whether the dendritic localization is involved in the local regulation of synaptic plasticity and higher-order brain functions.

The GO category “elongation factor” included *Eef1a1*, *Eef1b2*, and *Eef2* mRNAs. Many translational regulators are known to be recruited to RNA granules as proteins and transported to dendrites [6,196]. In contrast, the above elongation factors are recruited as mRNAs and may be translated locally in dendrites. It is interesting that, among several steps of translation such as initiation, elongation, and termination, the factors involved in the elongation step were selectively enriched in dendritic mRNAs. Translation initiation steps are mainly regulated by phosphorylation and dephosphorylation of the initiation factors. For example, phosphorylation of eIF4E promotes the initiation step, whereas phosphorylation of eIF2α in stress conditions inhibits translation initiation. In contrast, in the elongation step, the exchange between GTP and GDP forms of eEF1a and eEF2 is a key regulatory mechanism [207]. In addition, control of the amount of elongation factors may be important for the regulation of elongation. This may be related to the fact that decreased expression levels of Eef1a1 and Eef1b2 are associated with Alzheimer’s disease and intellectual disability, respectively [67,69]. Increasing concentrations of elongation factors at specific timings and locations mediated by local translation could be important for local synaptic regulation.

### 2.3. Expected GO Categories of the Catalogued Dendritic mRNAs: “Synapse”, “Dendritic Spine”, and “Ionotropic Glutamate Receptor Complex”

The other GO categories “synapse”, “dendritic spine”, and “ionotropic glutamate receptor complex” included mRNAs encoding postsynaptic proteins such as Arc, Kcnab2, Crtc1, Ncs1, Camk2a, Cdk16, Shank1, Homer2, Dlg2, Dlg4, Dlgap3, Sipa1l1, and Psd3 (Figure 2b,c). Most of these are known to play key roles in synaptic plasticity and higher-order brain functions (Supplementary Table S1). Arc accumulates at inactive synapses to prevent their enhancement and is involved in memory formation and neuronal diseases such as fragile X syndrome and Alzheimer’s disease [21,22,23,24,25,26]. Kcnab2, a potassium voltage-gated channel subunit, interacts with Shank3, and mice deficient in Kcnab2 exhibit amygdala hyperexcitability and impaired associative learning and memory [92,93]. Crtc1 is a CREB-regulated transcription factor that is transported from the synapse to the nucleus in response to late-phase LTP (L-LTP), where it enhances spatial memory and memory consolidation/reconsolidation, and its deficiency in mice showed depression-like behaviors and impaired memory formation [44,45,46,47,48,49]. Ncs1 is a calcium-binding protein involved in short- and long-term synaptic plasticity, dopaminergic signaling, learning, and memory, and its dysfunction leads to various neuronal diseases such as schizophrenia, bipolar disorder, Parkinson’s disease, and fragile X syndrome [97,98,99]. Camk2a is a calcium/calmodulin-dependent protein kinase subunit that plays key roles in synaptic plasticity, AMPA receptor transmission, LTP, and long-term memory formation, and its dysfunction underlies neuropsychiatric disorders such as schizophrenia, epilepsy, and fragile X syndrome [35,36,37,38,39,40,41,42]. Cdk16, a cyclin-dependent kinase, is responsible for dendrite development [43]. Shank1, Homer2, Dlg2, and Dlg4 are scaffold proteins of excitatory postsynaptic density. Shank1 regulates spine morphology and neurotransmission and is associated with ASD and neuropsychiatric disorders [156,157,158,159,160]. Homer2 interacts with dendritic spine actin regulators such as Cdc42 and drebrin, enhances cell surface expression of NMDA receptors, and is involved in spine maturation. Homer2 also interacts with amyloid precursor protein to inhibit Aβ production [86,87,88,89]. Dlg2 is associated with neurodevelopmental disorders, its deficiency in mice causes LTP impairment and hypersocial behavior, and its overexpression improves Aβ-mediated cognitive dysfunction [56,57,58,59,60]. Dlg4, also known as PSD-95, plays key roles in synaptic plasticity, spine growth, and AMPA and NMDA receptor regulation, and its dysfunction underlies neuropsychiatric disorders such as schizophrenia and autism [61,62]. Dlgap3, also known as SAPAP3, binds to Dlg4, regulates mGluR5-driven AMPA receptor trafficking, and its deficiency in mice exhibits obsessive–compulsive-disorder-like behaviors and altered ultrasonic vocalizations [63,64,65,66]. Sipa1l1 is a Rap GTPase-activating protein (GAP) involved in spine morphogenesis, homeostatic synaptic plasticity, learning, and memory, and its expression is altered by Aβ treatment [161,162,163,164,165,166,167,168,169,170,171,172,173]. Finally, Psd3 is a guanine nucleotide exchange factor of ADP-ribosylation factor 6 (Arf6), but its neuronal function is unknown. Thus, these dendritic mRNA-encoded proteins are implicated in synaptic plasticity and learning and memory, well-known roles of local translation in dendrites.

Of note, Homer2 and Psd3 were also classified as proteins that contain membrane-bound pleckstrin-homology (PH) domain in the GO enrichment analysis, although the Benjamini value (0.0535) did not reach significance. The GO categories of “PH domain” and/or “PH-like domain” included Homer2, Psd3, Psd, Plekhm2, Sptbn2, and Arhgap25. Compared with soluble proteins that diffuse easily in the cytosol, membrane-associated proteins are difficult to diffuse freely due to physical obstacles and electrostatic interactions within the membrane [208]. PH domains bind to phosphoinositides, which provide such electrostatic interactions [208,209]. Therefore, local translation of membrane-associated proteins appears to be an effective way to deliver low-mobility proteins at the required time and place.

## 3. mRNAs Bound to and Regulated by RNA-Binding Proteins of RNA Granules

RNA-binding proteins in RNA granules play a variety of roles in regulating mRNA stability, localization, and translation in neurons. Among them, we focus on five RNA-binding proteins: fragile X mental retardation protein (FMRP), RNG105/Caprin1, Ras-GAP SH3 domain binding protein (G3BP), cytoplasmic polyadenylation element binding protein 1 (CPEB1), and staufen double-stranded RNA binding proteins 1 and 2 (Stau1 and Stau2), of which associated mRNAs have been relatively extensively identified, and as summarized below, their knockout (KO)/knockdown (KD) in rodents are known to affect neural functions and higher-order brain functions, including memory formation.

### 3.1. Effects of RNA-Binding Proteins of RNA Granules on Higher-Order Brain Functions

FMRP is encoded by the *Fmr1* gene, in which mutations cause fragile X syndrome, a mental retardation disorder. FMRP regulates protein-synthesis-dependent synaptic plasticity, because Fmr1 KO mice exhibited enhanced metabotropic glutamate receptor (mGluR)-dependent long-term depression (LTD) that requires protein synthesis [210,211,212]. Although controversial, several studies reported the impact of FMRP on memory formation. Contextual fear memory in *Fmr1* KO mice was both impaired [213,214,215] and unaffected [216]. Spatial memory in the Morris water maze (MWM) in Fmr1 KO mice was also both impaired [217,218] and unaffected [213,219,220].

RNG105/Caprin1 is responsible for the formation and/or maintenance of dendrites and synaptic connections [221] and is essential for long-term memory formation. Even a slight deficiency of RNG105/Caprin1 in heterozygous mice impaired reversal learning in spatial learning tasks [222]. A more severe deficiency of RNG105/Caprin1 in forebrain-specific cKO mice significantly impaired long-term memory in both spatial MWM and contextual-fear-conditioning tasks [16].

G3BP plays a central role in the assembly of stress granules (SGs) in response to various types of stress [223,224]. G3BP1 KO mice showed impaired spatial working memory in the Y-maze test, but they had normal acquisition of nonspatial long-term memory in the passive avoidance test [225].

CPEB1 binds to cytoplasmic polyadenylation elements (CPEs) within the 3’UTR of mRNAs and mediates cytoplasmic polyadenylation of mRNAs to promote translation [226]. CPEB1 regulates synaptic plasticity as indicated by a deficit in LTP in CPEB1 KO mice [227]. CPEB1 KO mice showed normal acquisition and retention of MWM spatial memory and fear-conditioned contextual memory but reduced memory extinction in these tests [228].

Staufen is well known for its role in mRNA localization during *Drosophila* embryo development, whereas its orthologs are known to regulate synaptic plasticity in rodents: Stau1 and Stau2 are required for L-LTP and LTD, respectively [229,230]. Although Stau1 KO mice showed no abnormalities in memory formation [231], Stau2 deficiency affected several types of memory. Stau2 KD rats exhibited impairment in spatial working memory in a delayed matching to place task in the MWM and delayed nonmatching to place task in an eight-arm radial maze [232]. In addition, Stau2 KD rats showed deficits in temporal association memory in a trace fear conditioning task and spatial association memory in an inhibitory avoidance task [232]. Downregulation of Stau2 in mice displayed deficits in discriminating different spatial contexts in the Barnes maze task [233].

### 3.2. Methods for Identifying RNA–Protein Interactions

To better understand the mechanistic basis of the RNA-binding-protein-mediated regulation of biological functions, including brain functions, it is important to identify target mRNAs for RNA-binding proteins. Various methods have been developed for this purpose [234,235].

In one method, RNA–protein direct binding in vitro has been analyzed using systematic evolution of ligands by exponential enrichment (SELEX). In SELEX, binding cycles between RNA-binding proteins and RNA pools are repeated to select high-affinity RNAs, and these RNAs are amplified by reverse transcription PCR, which detects RNAs that bind to proteins with high probability [236,237,238].

Second, RNA–protein association in cells and tissues has been identified with RNA-immunoprecipitation (IP), UV-crosslinking IP (CLIP), and electrophoretic mobility shift assay (EMSA). In CLIP, UV irradiation of cells or tissues generates covalent bonds between RNA and RNA-binding proteins when they are in close proximity to each other, followed by co-IP of the RNA with the RNA-binding proteins [239,240,241]. Other modified CLIP methods have been developed, such as high-throughput sequencing CLIP (HITS-CLIP) and photoactivatable ribonucleoside-enhanced CLIP (PAR-CLIP). HITS-CLIP is a combination of CLIP and high-throughput sequencing that enables comprehensive identification of associated RNAs [240,241]. In PAR-CLIP, RNA is labeled with photoreactive nucleoside analogs, and cells are irradiated with 365 nm UV. As a result, crosslinking efficiency is improved compared with conventional CLIP [240]. In EMSA, labeled RNA pools are incubated with cell and tissue extracts, and the binding of RNA to proteins can be detected by reduced mobility of the RNA in gel electrophoresis [242,243]. 

Third, RNA-protein association has also been identified using gene overexpression, KD, and KO of RNA-binding proteins in cells and animals. These altered expression levels affect the stability, localization, and translation efficiency of the target mRNAs depending on the effect of the RNA-binding proteins on the mRNAs. Differential analysis of these changes compared with control cells and animals, using microarrays, RNA-seq, ribosome footprints, and their combination with the isolation of specific regions of cells, can identify mRNAs regulated by RNA-binding proteins. There are also various other methods to analyze RNA-protein associations, such as sequence- and structure-based methods using computational approaches [235].

Using these methods, target mRNAs of RNA-binding proteins in RNA granules have been identified. We comprehensively list the target mRNAs of FMRP, RNG105/Caprin1, G3BP, CPEB1, Stau1, and Stau2 and summarize the effects of the RNA-binding proteins on the mRNAs (Table 1 and Supplementary Table S2a–e). Furthermore, we compared the list of the dendritic mRNAs (Supplementary Table S1) with the target mRNAs of the RNA-binding proteins (Supplementary Table S2a–e) to find dendritic mRNAs that are associated with and/or regulated by FMRP, RNG105/Caprin1, G3BP, CPEB1, Stau1, and Stau2 (Supplementary Table S1). In the following subsection, we discuss how the RNA-binding proteins regulate their target mRNAs during mRNA localization and local translation in dendrites.

### 3.3. Effects of the RNA-Binding Proteins on mRNA Regulation

FMRP target mRNAs have been identified in many studies and are listed in Supplementary Table S2a [244,245,246,247,248,249,250,251,252,253,254,255,256,257,258,259,260,261,262,263,264,265,266,267,268,269,270,271,272,273,274,275,276,277,278,279,280,281,282,283,284,285,286,287,288,289,290,291,292,293,294,295,296,297,298,299,300,301,302,303,304]. FMRP has various effects on mRNA regulation such as mRNA expression, stability, localization, and translation, among which the major role of FMRP is translational regulation, as shown in a number of studies (Supplementary Table S2a). FMRP represses translation through multiple mechanisms in the basal state, but the repression is relieved by dephosphorylation of FMRP upon mGluR stimulation (references in Supplementary Table S2a). CPEB1 also regulates translation of its target mRNAs. The CPEB1 target mRNAs are listed in Supplementary Table S2d [305,306,307,308,309,310,311,312,313,314,315,316,317,318,319,320,321,322,323,324,325,326,327,328,329,330,331,332,333,334]. CPEB1 represses translation in the basal state but is phosphorylated upon synaptic stimulation, thereby polyadenylating its target mRNAs and promoting translation (references in Supplementary Table S2d). In neurons, both FMRP and CPEB1 are localized in dendrites and in/near spines [335,336,337,338]. Given that many of the 78 dendritic mRNAs have been identified to be associated with FMRP and CPEB1 (i.e., 28 with FMRP and 11 with CPEB1 (Supplementary Table S1)), FMRP and CPEB1 may regulate activity-dependent translation of dendritic mRNAs in dendrites near spines.

In contrast, RNG105/Caprin1, for which target mRNAs are listed in Supplementary Table S2b [16,221,339,340,341,342], is involved in mRNA localization. RNG105/Caprin1-containing RNA granules are localized throughout the proximal to distal regions of dendrites [221,339]. RNG105 cKO reduced the dendritic localization of many (46 of 78) of the dendritic mRNAs (Supplementary Table S1) [16]. This suggests that RNG105 plays an important role in mRNA localization to proximal and distal dendrites to achieve local translation, which may be necessary for the formation of long-term memory. In addition to mRNA localization, RNG105/Caprin1 may play a role in spatially biasing translation to RNA granules: overexpression of RNG105/Caprin1 in cells locally increased translation in/near RNA granules but suppressed translation in the cytoplasm [343]. Staufen also regulates mRNA localization in addition to participating in mRNA decay, known as Staufen-mediated mRNA decay. The target mRNAs of Staufen are listed in Supplementary Table S2e [230,312,344,345,346,347,348,349,350,351,352,353,354,355,356,357,358,359,360]. Of the 78 dendritic mRNAs, 44 were associated with Stau1 and/or Stau2 (Supplementary Table S1). Although both Stau1 and Stau2 show dendritic localization in neurons, Stau2 is localized to more distal dendrites than Stau1 [361]. It was reported that some dendritic mRNAs are restricted to proximal dendrites, but others are localized not only to proximal but also distal dendrites [15]. Given the different distributions along the proximal–distal dendritic axis of RNG105/Caprin1, Stau1, and Stau2, these RNA-binding proteins may regulate mRNA localization to the appropriate region of dendrites.

The association of G3BP with mRNA has been analyzed in several studies, including comprehensive identifications of G3BP-associated mRNAs (Supplementary Table S2c) [362,363,364,365,366,367,368,369,370,371,372]. However, no G3BP-associated mRNAs have been found in the 78 dendritic mRNAs (Supplementary Table S1). The dendritic mRNAs were identified in the hippocampus [15,16,17], but fibroblasts were used to comprehensively identify G3BP-associated mRNAs [370,371]. This difference in mRNA sources may be a reason for the absence of G3BP-associated mRNAs in dendritic mRNAs. Alternative reasons may come from the G3BP distribution pattern in neurons: although G3BP is localized to dendrites in addition to the soma in primary cultured neurons [221], G3BP is almost restricted to the soma in hippocampal CA1 pyramidal neurons in vivo [225]. Thus, G3BP may preferentially target somatic mRNAs rather than dendritic mRNAs in hippocampal pyramidal neurons. It should also be noted that G3BP is a key protein that facilitates the assembly of SGs, of which many components are shared with neuronal RNA granules. SGs are formed only under stress, but neuronal RNA granules are always present, even when neurons are unstressed. Arsenite treatment of neurons that already have RNA granules has been reported to induce the assembly of another population of granules containing RNA-binding proteins such as G3BP, TIAR, and FMRP [129,373,374]. Thus, stress-induced granules may differ from granules present in unstressed neurons. In this case, G3BP may primarily target mRNAs in SGs rather than regulating dendritic mRNAs for neuronal synaptic plasticity.

Each RNA-binding protein has its own functions that regulate the expression, stability, localization, and translation of its target mRNAs. In addition to executing these intrinsic functions, RNA-binding proteins in RNA granules may also play roles in regulating mRNAs through controlling the condensation and dissolution of RNA granules. Recently, it has been revealed that RNA granules are formed by liquid–liquid phase separation and their liquid-like and solid-like states can be dynamically controlled [375,376,377,378,379,380,381]. Each RNA-binding protein exhibits different dynamics in RNA granules, and their combinations in RNA granules affect the physical properties of RNA granules and the translation efficiency in/near RNA granules [343]. Understanding the effects of physical properties and condensation/dissolution behavior of RNA-binding proteins on mRNA regulation may be also important to better understand the mechanistic basis of mRNA localization and local translation in neurons.

## 4. Future Perspectives

The functions of RNA-binding proteins have been revealed in many studies. In addition, their target and dendritic mRNAs have been identified, and the functions of the proteins encoded by these mRNAs have been elucidated. However, there is limited understanding of the physiological relevance of locally increasing the concentration of mRNA-encoded proteins through mRNA localization and local translation. Exceptionally, *Camk2a* mRNA localization to dendrites has been reported to be required for L-LTP and long-term memory formation. This was revealed by the deletion of the 3’UTR, which caused a loss of dendritic localization of *Camk2a* mRNA but did not affect its translation [382]. However, the relevance of such local regulation of most other dendritic mRNAs to synaptic plasticity and brain functions remains elusive. To determine this, high-throughput methods cannot be used, and each mRNA has to be analyzed individually. Nevertheless, recent advances in comprehensive identification technology have identified a large number of candidates for dendritic mRNAs and RNA-binding protein target mRNAs, and selective ones need to be prioritized. In this review, we have catalogued and selected mRNAs that are localized to dendrites and regulated by RNA-binding proteins in RNA granules. A future challenge in this research field is to elucidate the biological and physiological relevance of local regulation of gene expression, which can be solved by artificially manipulating local regulation, such as altering mRNA localization without affecting translation efficiency and affecting RNA granule assembly without losing essential functions of RNA-binding proteins. We hope this review will help researchers in this field find gemstones in the wilderness.

## Figures and Tables

**Table 1:** Summary of Supplementary Tables S1 and S2.

Table	Title	Content		
Supplementary Table S1	Dendritic mRNAs and their associated RNA-binding proteins	Candidate dendritic mRNAs commonly identified in studies that used RNA sequencing (RNA-seq) (Cajigas et al., 2012; Ainsley et al., 2014; Nakayama et al., 2017) are listed, and the general and neuronal functions of the encoded proteins are summarized. Association of these mRNAs with RNA-binding proteins of RNA granules—FMRP, RNG105/Caprin1, G3BP, CPEB1, Stau1, and Stau2—are indicated.		
Supplementary Table S2a–e ^1,2^	Target mRNAs of RNA-binding proteins of RNA granules	mRNAs associated with and regulated by the RNA-binding proteins of RNA granules are listed. Effects of the RNA-binding proteins on the mRNAs are summarized.		RNA-binding proteins
			2a	FMRP
			2b	RNG105/Caprin1
			2c	G3BP
			2d	CPEB1
			2e	Staufen, Stau1, Stau2

^1^ “Target mRNA (Gene Name)” indicates the gene name shown in the references. “Gene Symbol” indicates the official gene symbol in *Mus musculus*. ^2^ Because the comprehensive analyses identified a large number of target mRNAs, the identified mRNAs are not concretely listed but indicated by asterisks (*). However, some comprehensive studies analyzed particular mRNAs more specifically. In such cases, those particular mRNAs are listed separately. Abbreviations: FMRP—fragile X mental retardation protein; RNG105—RNA granule protein 105; G3BP—Ras-GAP SH3 domain binding protein; CPEB1—cytoplasmic polyadenylation element binding protein 1; Stau1 and Stau2—staufen double-stranded RNA binding proteins 1 and 2.

## Supplementary Materials

The following are available online at https://www.mdpi.com/2218-273X/10/2/167/s1, Table S1: Dendritic mRNAs and their associated RNA-binding proteins. Table S2a: FMRP target mRNAs. Table S2b: RNG105/Caprin1 target mRNAs. Table S2c: G3BP target mRNAs. Table S2d: CPEB1 target mRNAs. Table S2e: Staufen, Stau1 and Stau2 target mRNAs.
